# The Impact of the COVID-19 Pandemic on the Level of Physical Activity, Emotional State, and Health Habits of Women in Late Pregnancy and Early Puerperium

**DOI:** 10.3390/ijerph20031852

**Published:** 2023-01-19

**Authors:** Daria Kołomańska-Bogucka, Natalia Pławiak, Agnieszka I. Mazur-Bialy

**Affiliations:** 1 Department of Biomechanics and Kinesiology, Faculty of Health Science, Jagiellonian University Medical College, Skawińska 8, 31-066 Krakow, Poland; 2 University Hospital in Krakow, Jakubowskiego 2, 30-688 Krakow, Poland

**Keywords:** physical activity during pregnancy, health behaviors at pregnancy, postpartum depression, pregnancy during pandemic

## Abstract

The aim of the study was to determine the impact of the Covid-19 pandemic on the level of physical activity in the last trimester, the risk of developing postnatal depression, and general health habits in late pregnancy and the early postpartum period. Methods: The study population was women 1–8 days postpartum. Participants were divided into three groups depending on when they were recruited: (1) prepandemic (Ppan: n = 252, December 2019–March 2020), (2) COVID1 group (Cov1: n = 262, May 2020–September 2020), and (3) COVID2 group (Cov2: n = 226, June 2021–September 2021). The Ppan group included women from before the pandemic. The Cov1 group included patients after some restrictions were lifted. The Cov2 group included women after vaccinations became available. Research tools included a demographical questionnaire (age, education, childbirth details), the Pregnancy Physical Activity Questionnaire (PPAQ), the Edinburgh Postnatal Depression Scale (EPDS), and the Health Behavior Inventory (IZZ). Results: Regression analysis showed that regardless of other variables, women who gave birth during the pandemic spent less energy on total physical activity compared to the prepandemic group (Cov1: β = −18.930, 95%CI: −36.499 to −1.361; Cov2: β = −26.527, 95%CI: −44.322 to −8.733). We also found that as the risk of depression increased, engagement in general health habits decreased during the pandemic. Conclusions: The Covid-19 pandemic decreased the level of some subdomains of physical activity in pregnant women, with a general negative correlation between emotional state and healthy habits.

## 1. Introduction

The COVID-19 pandemic was formally announced by the World Health Organization (WHO) on 11 March 2020 [1] and led to the introduction of many restrictions around the world. The first case in Poland was identified on 4 March 2020, with a state of epidemic being announced on 20 March. With it came significant restrictions in daily living, e.g., school, park, and forest closures, along with travel bans. These restrictions began to ease at the end of April 2020 with gyms, fitness clubs, and swimming pools reopening by June 2020. However, due to the differences in infection rates in different regions of Poland, three restriction zones were created, i.e., green (no restrictions), yellow (mild restrictions), red (heaviest restrictions), and were updated weekly based on a running 14-day average of infection data [2].

While maternity wards did not reduce their activity during the pandemic, some restrictions were introduced, including a ban on visits and families being present for deliveries. These were not universal, obligatory rules for hospitals, so limited visitations, e.g., one person for a brief period, along with teleconsultations, were sometimes possible [3,4]. The state of epidemic ended in Poland on 16 May 2022 [5]. Considering the above limitations, the pandemic clearly had a potentially significant impact on the lives of pregnant and postpartum women. There are already several studies examining the impact of the pandemic on the psychophysical health of pregnant women [6,7,8,9,10] and the postpartum period [11,12,13]. However, to our knowledge, there are currently no studies evaluating changes caused by the pandemic to the combined physical activity, emotional state, and general health behaviors in women during the perinatal period.

Research shows that the Covid-19 pandemic had a negative impact on mental health [14,15,16,17], especially among pregnant and postpartum women [18,19,20,21] who experienced increased anxiety, depression, relationship strain, and social isolation [20]. Discomfort during hospital visits was also associated with an increased likelihood of developing depression [22]. Moreover, the frequency of depression was significantly higher in pregnant women who tested positive for Covid-19 compared to those who tested negative or had never been tested [23]. Yan et al. [24] noted higher rates of depression in multiparous women, as well as in pregnant women, in their first and third trimesters. Depressive disorders are known to increase the incidence of pre-eclampsia, caesarean section, and episiotomy [25]. Women suffering from prenatal depression have higher levels of anxiety and fear of childbirth [26]. Unfortunately, mental health problems also increase the risk of suicide for future mothers [27]. Untreated depression may result in preterm labor, perinatal complications, increased cortisol levels in the newborn, and developmental disorders in children [28,29]. Depression is treated with psychotherapy and/or pharmacotherapy; however, women tend to opt for non-pharmacological options [30], such as music therapy [31], massages [32], behavioral therapy [33], and family support [34]. When properly utilized, physical activity, such as walking, yoga, resistance training, and water exercises, can significantly reduce the risk of developing perinatal and postpartum depression and promote a healthy lifestyle [35,36,37].

WHO advises that both pregnant and puerperal women should minimize the amount of time spent sitting, emphasizes the health benefits of low-intensity activity, and recommends at least 150 min per week of physical activity [38,39]. Although regular physical activity during pregnancy promotes good mental and physical health [40], many women still significantly reduce their activity [41]. The main causes of inadequate activity are fatigue, work, pain, as well as lack of time, motivation, and support [42]. The Covid-19 pandemic caused many changes in the daily lives of pregnant women, especially their physical activity [43]. Hillyard et al. [44] showed that during the pandemic, only 23% of women (compared to 47% before the pandemic) met the guidelines for optimal physical activity, and as many as 69% feared leaving home. In addition, women in urban areas were significantly less active [45].

The aim of the study was to determine the impact of the Covid-19 pandemic on the level of physical activity in the last trimester, the risk of developing postnatal depression, and women’s general health habits in late pregnancy and the early postpartum period. We hypothesize that the Covid-19 pandemic significantly reduced physical activity in pregnant women, increased the risk of developing postpartum depression, and improved the general health habits of women in late pregnancy and the early postpartum period.

## 2. Materials and Methods

### 2.1. Participants

The study included 740 obstetric patients from three Cracow hospitals who were between 1–8 days postpartum. Patients were divided into three groups: prepandemic (Ppan) i.e., from before the pandemic, between December 2019 and March 2020; COVID1 (Cov1) from between May and September 2020; and COVID2 (Cov2) from when vaccines were introduced, between June and September 2021. The exact characteristics, course, and sample selection are presented in our previous publication [46].

### 2.2. Measures

The research tools began with a demographics questionnaire (e.g., age, place of residence, and education). After the Covid-19 pandemic began, we added questions about the psychophysical condition and lifestyle of pregnant women compared to the prepandemic period. Patients were able to answer each question using a Likert scale from 0 (no impact) to 5 (total impact). We used the 35-question Polish version [47] of the Pregnancy Physical Activity Questionnaire (PPAQ) [48] to assess the level of physical activity in the last trimester and calculate the average weekly energy expenditure for total activity, sport activity, physical exercise, passive rest, movement, home activities, and work. We stratified physical activity into sedentary, low, moderate, and high intensity [47,48]. The Intraclass Correlation Coefficient evaluating the level of reliability was 0.75 for total activity. The level of reliability for activity by intensity was as follows: sedentary 0.70, light 0.53, moderate 0.85, vigorous 0.86. We also measured by type: sports/exercise 0.83, transportation 0.59, household/caregiving 0.89, work activity 0.86 [49]. The PPAQ scale is often used to assess physical activity during pregnancy [50]. We used the Polish version [51] of the Edinburgh Postnatal Depression Scale (EPDS) [52] to screen for postpartum depression. The EDPS scale consists of 10 statements to assess well-being over the past 7 days. Each question can be answered on a scale from 0 to 3, with a maximum score of 30. A score of 12 points or higher qualifies as major depression [51,52]. The Cronbach’s alpha reliability index for EDPS is 0.91 (Polish version) [53] and is often used to assess the risk of developing postpartum depressive disorders [54,55,56,57]. Patients’ health habits were assessed using the Health Behavior Inventory (IZZ), which consists of 24 statements regarding various types of general health-related behaviors during the last year. The final score follows a sten scale and represents the level of general health-promoting behavior among patients (1–4: low, 5–6: average, and 7–10: high). It considers eating habits, health practices, prevention methods, and positive mental attitudes, where each question is scored from 1 (almost never) to 5 (almost always). Higher sum scores correspond with better health behaviors. The IZZ scale has separate norms for men and women. The Cronbach’s alpha reliability index for IZZ is 0.85 [58].

Participation in the study was voluntary and all study participants gave informed consent. The study was conducted in accordance with the Helsinki Declaration and approved by the Jagiellonian University Bioethics Committee (1072.6120.251.2019, 24 October 2019).

### 2.3. Statistical Analysis

Statistical analysis was performed using PS Imago Pro 7 and PS Imago Pro 8. Normality was assessed by Shapiro-Wilk test. The Kruskal-Wallis test was used to assess the relationship between three independent groups, followed by the Wilcoxon test with Bonferroni correction. We also performed chi-square tests of independence and Spearman’s rank correlation. The aim of the correlation was to assess the relationship between depressive disorders and health behaviors on the type and intensity of physical activity. Based on the obtained results, we can show how physical activity changed due to depressive disorders and general health behaviors. Linear regression was performed to identify factors influencing physical activity. In the regression analysis, we used 0–1 coding, in which categories coded as 0 were considered reference categories (ref.). Confounding factors were selected on the basis of statistical analyses and available literature. Multivariate linear regression analysis was performed to examine the associations between total physical activity and its subdomains, i.e., type and intensity, during the pandemic, risk of developing depression, health behaviors, and other factors. The controlled variables in the regression analysis included marital status, place of residence, number of deliveries, education, planning the current pregnancy, complications during pregnancy, age (years), delivery at term, and type of delivery. The effect of the size of the associations is represented by the B coefficient. The value of *p* < 0.05 was considered statistically significant

## 3. Results

The majority of survey responders were married, highly educated women living in large cities. There were more vaginal births than caesarean sections, independent of the study period. Most women were primiparous before (Ppan) and during the later stage of the pandemic (Cov2), while in the early pandemic (Cov1) most were multiparous. Patients had decreased total physical activity during the pandemic, Cov1 also had significantly reduced mobility, and Cov2 had significantly reduced household activities. Detailed results are presented in our previous work [46].

### 3.1. Physical Activity Based on Intensity Level

The results regarding the intensity of activity showed that women in the third trimester of pregnancy led a more sedentary lifestyle before the pandemic than during the pandemic (Ppan.: Me: 15.04, Q1–Q3: 7.35 −29.40 MET; Cov1: Me: 7.35, Q1–Q3: 4.69–18.90 MET; Cov2: Me: 7.35, Q1–Q3: 4.69; 18.90 MET). Women giving birth before the pandemic were also characterized by higher levels of light physical activity (Ppan: Me: 108.15, Q1–Q3: 68.77–155.38 MET; Cov1: Me: 91.40, Q1–Q3: 60.54–132.18 MET; Cov2: Me: 88.88, Q1–Q3: 51.44–120.35 MET). In the case of moderate activity, the highest expenditure was shown in participants from the Cov1 group; however, no significant differences were found. The least energy was devoted to vigorous physical activity in all groups (Table 1). Analyses of changes by activity type were presented in our previous article [46].

### 3.2. Risk of Postpartum Depression and Women’s Health Behavior

The probability of developing postpartum depression was assessed using the EPDS scale. The majority of women across all groups were not found to be at risk of developing postnatal depressive disorders (PPan: 77.92%, Cov1: 78.91%, Cov2: 83.63%). The level of health-related behaviors was determined using the IZZ questionnaire. The analysis shows that Ppan (85.0), Cov1 (86.0), and Cov2 (84.5) periods had similar general health habits (Table 2). In addition, in Appendix A, we added detailed information on the correlations between depressive disorders and participants’ health behaviors. These analyses show that regardless of the study period, the risk of developing postpartum depressive disorders in patients increased when general health behavior decreased (Appendix A).

### 3.3. Risk of Postpartum Depressive Disorders and Physical Activity

Based on the results of the EPDS, we divided the patients into those at risk of developing postpartum depression (EPDS ≥ 12) and those not at risk (EPDS < 12). We observed a positive correlation between increased passive rest (Ppan: r = 0.337, *p* = 0.018) and sedentary activities (Ppan: r = 0.399, r = 0.004), and the risk of developing depressive disorders (Table 3) only in prepandemic women. We conducted identical analyses for patients in the no risk group (Appendix A).

### 3.4. Health Behavior and Physical Activity

A positive correlation between sport activities and general health behaviors was found among all groups, with the strongest correlation observed in Ppan (r = 0.245, *p* < 0.001; Cov1: r = 0.162, *p* = 0.014; Cov2: r = 0.170, *p* = 0.012). In Cov2, the level of health behaviors was negatively correlated with total physical (r = −0.154, *p* = 0.024), home (r = −0.189, *p* = 0.005), and moderate physical activity (r = −0.137, *p* = 0.044). Ppan showed a tendency to reduce general health behavior and increase sedentary activity (r = −0.128, *p* = 0.060). Nevertheless, their health habits improved with increased vigorous physical activity (r = 0.164, *p* = 0.016). We did not notice similar relationships in the other groups (Table 4).

### 3.5. Behavioral and Lifestyle Changes in Women during the COVID-19 Pandemic

In order to show changes in lifestyle during the pandemic, we added questions to the original questionnaire regarding the level of anxiety, stress, fear of infection, home isolation, limiting physical and social activity, and accepting the ban on family-accompanying deliveries. These may affect general health behaviors and the incidence of depressive disorders. Over 90% of Cov1 and 80% of Cov2 showed increased stress, anxiety, fear of infection, and home isolation. In addition, nearly 88% of Cov1 and 75% of Cov2 reduced their physical activity (Appendix A, Table A3 and Table A4). We listed correlations between the above variables and depressive disorders and health behaviors (Appendix A, Table A5 and Table A6).

### 3.6. Physical Activity of Women at Risk of Depression—Multivariate Linear Regression

In order to analyze the associations between physical activity—type and intensity—and depression, health behaviors, and other confounding factors during the pandemic, we performed a multivariate linear regression analysis. We only observed that patients diagnosed with the risk of developing depressive disorders significantly reduced their mobility activities during the pandemic. Nevertheless, this relationship was obtained only among women giving birth in the early phase of the pandemic (Cov1: β = −11.166, 95%CI: −21.624 to −0.690, Model 1). We did not find similar relationships in other cases (Table 5).

We performed a multivariate linear regression on the intensity of physical activity. During the pandemic, patients at risk of depression also experienced changes in the level of light physical activity compared to the prepandemic group. However, the reduction in light physical activity occurred only in late pandemic participants (Cov2: β = −27.318, 95%CI: 52.801 to −1.836). We did not observe similar relationships in the case of sedentary, moderate, and vigorous physical activity (Table 6).

## 4. Discussion

The aim of the study was to determine the impact of the Covid-19 pandemic on the level of physical activity in the last trimester, the risk of developing postnatal depression, and women’s general health habits in late pregnancy and the early postpartum period.

We observed that women at risk of depression decreased movement (Cov1) and light activities (Cov2) during the pandemic, independent of variables, such as the risk of developing depressive disorders, health habits, age, place of residence, level of education, marital status, number as well as types of births, pregnancy planning, complications, and timeliness of delivery. Current health habits did not influence the likelihood of developing postpartum depression. However, developing depression caused a reduction in the level of general health habits, independent of the study period. Mental health and health behavior were also negatively affected by fear of infection, stress, and anxiety, as well as limiting physical and social activity.

Among Polish women, the amount of energy expenditure for sedentary physical activity varied by trimester: first trimester: 40.26 MET, second trimester: 38.43 MET, third trimester: the values may range from 29.4 to 61.4 MET [59,60,61]. In our study, third trimester patients devoted between 88.88 to 108.15 MET to sedentary activity. Women giving birth before the pandemic devoted more of their energy to a sedentary lifestyle. Studies show that the trimester of pregnancy also affects the level of light and moderate activity (from 79.78 to 109.8 MET and from 23.7 to 40.13 MET, respectively) [59,60,61]. Interestingly, our study indicates that while patients during the pandemic had less sedentary activity and low general activity, they did not significantly increase their moderate and vigorous activity. Despite this, over 87% of Cov1 and nearly 75% of Cov2 admitted that the pandemic had limited their current activity. This percentage was higher than in other publications [44,62]. In contrast, Whitaker et al. [63] reported that 22% of pregnant women completely gave up physical activity during the pandemic.

Affective disorders during pregnancy and the peripartum period are frequent and consequential [64]. However, fear of infection (in baby and self) [65,66], discomfort associated with hospital visits, and the pandemic [22] may significantly increase the risk of developing depression in new mothers. Between 13% and 75% of women suffered from postpartum depression [65,66,67,68,69] during the pandemic. Interestingly, our data show that the percentage of emotional problems in women was 22% before the pandemic (Ppan), then it slightly decreased to 21% (Cov1) and 16% (Cov2). Despite this, nearly 92% of Cov1 and 85% of Cov2 feared infection. Abujilban et al. [70] indicated that sufficient knowledge about Covid-19 may be a protective factor against depression during pregnancy. A study by Motrico et al. [71] reported that between 33.3% and 47.2% of women during the pandemic experienced perinatal anxiety and depression. In addition, nearly 30% were diagnosed with high levels of anxiety and depressive disorders. Risk factors were the increased concerns about the financial effects of the pandemic [71].

Physical activity has a positive impact on the emotional state of pregnant women [20]. Meta-analysis by Nakamura et al. [72] indicates that physical activity during pregnancy minimizes the risk of developing postpartum depression. Gildner et al. [45] noted that reduced physical activity during the pandemic contributed to increased depressive disorders in pregnant women. Low physical activity before childbirth positively correlates with a higher risk of developing depression before and immediately after childbirth, and also 6 months postpartum [73]. Sánchez-Polán et al. [74] noted a 16% higher probability of developing depression in physically inactive pregnant women. The risk of postpartum depression may also be increased by a sedentary lifestyle and less household activity [75]. In our study, only among women from the prepandemic group at risk of depression, we observed that the risk of depressive disorders increases with passive rest and sedentary activities. During the pandemic, patients at risk of depression reduced mobility and light physical activity. Nevertheless, these changes depended on the period of the pandemic.

Davenport et al. [76] reported that moderate activity during pregnancy and the postpartum period helps mitigate emotional disturbances in women. Additionally, online training positively affects the general mental well-being of pregnant women [77]. Studies from the early pandemic showed that the majority of pregnant women tried to pay attention to their mental health [78]. In the same vein, our results demonstrate that 95% of patients in 2020 and 88% in 2021 believed that the pandemic caused them more anxiety and stress. The development of anxiety, depression, and stress in pregnant women is influenced by thoughts about the COVID-19 pandemic, fear for their own health and the health of the fetus, and the experience of abnormal somatic symptoms [79].

Women had similar levels of general health habits regardless of the study period. Similarly, Matvienko-Sikar et al. [80] did not find significant differences in health behaviors between pregnant women before or during the pandemic. In fact, Pope et al. [81] indicated that pregnant women were highly involved in healthy behaviors during the pandemic. We found that depressive disorders negatively impact general healthy behavior. Lobel et al. [82] indicated that stress significantly influences health behavior, specifically that stress in the perinatal period is positively correlated with smoking, caffeine consumption, and unhealthy eating. A balanced diet, vitamin supplementation, and exercise decreases stress [82]. However, Oeschle et al. [83] found that many pregnant women lack adequate knowledge about dietary recommendations (55%) and the importance of physical activity (48%). Lifestyle education results in improved pro-health behaviors [84]; we observed a positive correlation between energy expenditure devoted to sports, physical exercise, and health behaviors in all three groups (Ppan: *p* < 0.001; Cov1: *p* = 0.014; Cov2: *p* = 0.012).

### Strengths and Limitations

This study is a continuation of the results presented in our previous publication [46], which provides a detailed assessment of the lifestyle of women giving birth during the Covid-19 pandemic. An important strength of the current study is the long time scale [December 2019–September 2022], which makes it possible to compare the psychophysical conditions of women at different stages of the pandemic. Additionally, we added questions to the original questionnaire about changes to the psychophysical state during the pandemic compared to before. The current study used subjective questionnaires and relied on participant self-report. The study was conducted only in hospitals located in Cracow due to various pandemic restrictions, which potentially limits the generalizability of our results to the entire population.

## 5. Conclusions

Women in the third trimester of pregnancy during the pandemic devoted less energy to sedentary and light-intensity activities compared to before the pandemic. However, there were no significant changes in moderate activity. Additionally, patients avoided vigorous activity regardless of the study period, with a significant number blaming the pandemic for limiting their physical activity. These patients indicated that the pandemic also increased their stress and anxiety, conditions associated with an increased incidence of depression. However, while the pandemic was generally not shown to increase the risk of developing depressive disorders in pregnant and postpartum women, when there was an increased risk within a subgroup, there was also a subsequent decrease in general health habits. In addition, regardless of confounding factors, the pandemic has reduced mobility and light-intensity activities in women at risk for depression.

## Figures and Tables

**Table 1:** The intensity of physical activity in the studied women.

Parameters	Prepandemic Group	COVID1 Group	COVID2 Group	
Physical Activity by Intensity (MET)	(N = 252) Me (Q1; Q3)	(N = 262) Me (Q1; Q3)	(N = 226) Me (Q1; Q3)	*p* *
Sedentary	15.04 (7.35; 29.40) ^a,#^	7.35 (4.69; 18.90) ^a,#^	7.35 (4.69; 18.90)	0.044
Light	108.15 (68.77; 155.38) ^a,b,#^	91.40 (60.54; 132.18) ^a,#^	88.88 (51.44; 120.35) ^b,#^	<0.001
Moderate	32.96 (16.52; 62.17)	38.35 (17.43; 82.12)	31.85 (11.51; 68.02)	0.086
Vigorous	0.00 (0.00; 0.00)	0.00 (0.00; 0.00)	0.00 (0.00; 0.00)	1.00

Me—median; Q1—lower quartile; Q3—higher quartile; Prepandemic—before the pandemic (Dec 2019 to Mar 2020); COVID1—early in the pandemic (May to Sept 2020); COVID2—late in the pandemic (June to Sept 2021); ^#^ denotes significant difference between designated groups: ^a^—Prepandemic vs. COVID1, ^b^—Prepandemic vs. COVID2; MET-h/week—metabolic equivalent of task (average weekly energy expenditure: MET-hour/week); * based on Kruskal–Wallis.

**Table 2:** The level of depressive disorders and health behaviors in women.

Parameters	Prepandemic Group	COVID1 Group	COVID2 Group	
	(N = 252)	(N = 262)	(N = 226)	*p* *
EPDS [Me (Q1; Q3)]	7.0 (4.0; 11.0)	6.0 (3.0; 10.0)	6.0 (4.0; 9.0)	0.130
EDPS < 12 [n/%] EPDS ≥ 12 [n/%]	180/77.92 51/22.08	202/78.91 54/21.09	189/83.63 37/16.37	0.262
IZZ [Me (Q1; Q3)]	85.0 (77.75; 92.0)	86.0 (77.0; 94.0)	84.5 (77.0; 93.0)	0.90
IZZ [n/%] Low Moderate High	60/24.8 120/49.6 62/25.6	62/25.8 102/42.5 76/31.7	57/26.1 97/44.5 64/29.4	0.549

Me—median; Q1—lower quartile; Q3—higher quartile; Prepandemic—before the pandemic (Dec 2019 to Mar 2020); COVID1—early in the pandemic (May to Sept 2020); COVID2—late in the pandemic (June to Sept 2021); EPDS—Edinburgh Postnatal Depression Scale; IZZ—Health Behavior Inventory (pol. Inwentarz Zachowań Zdrowotnych); * based on Kruskal–Wallis test or chi-square test of independence.

**Table 3:** Spearman’s Rank Correlations between depressive disorders and physical activity—type and intensity of activity.

The Risk of Postpartum Depressive Disorders						
Group	Prepandemic		COVID1		COVID2	
Parameters	*p*	Spearman’s Rank	*p*	Spearman’s Rank	*p*	Spearman’s Rank
Activity by type						
Total physical activity	0.396	0.124	0.238	0.168	0.249	0.197
Sports activity and physical exercise	0.821	0.033	0.246	0.165	0.665	0.075
Passive rest	0.018	0.337	0.764	−0.043	0.503	0.115
Movement	0.128	0.220	0.132	0.214	0.137	0.252
Household activities	0.687	0.059	0.367	0.129	0.526	0.109
Occupational activity	0.928	−0.013	0.581	0.079	0.718	0.062
Activity by intensity						
Sedentary	0.004	0.399	0.646	−0.066	0.443	0.132
Light	0.759	0.045	0.230	0.171	0.924	0.016
Moderate	0.707	0.055	0.319	0.142	0.134	0.254
Vigorous	0.683	0.060	0.582	−0.079	0.191	−0.223

Statistical significance for *p* < 0.05; Spearman’s rank—Spearman’s rank correlation coefficient; EPDS ≥ 12; Prepandemic—before the pandemic (Dec 2019 to Mar 2020); COVID1—early in the pandemic (May to Sept 2020); COVID2—late in the pandemic (June to Sept 2021).

**Table 4:** Spearman’s Rank Correlations between general health behavior and physical activity—type of activity.

General Health Behaviors						
Group	Prepandemic		COVID1		COVID2	
Parameters	*p*	Spearman’s Rank	*p*	Spearman’s Rank	*p*	Spearman’s Rank
Activity by type						
Total physical activity	0.971	−0.002	0.088	−0.114	0.024	−0.154
Sports activity and physical exercise	<0.001	0.245	0.014	0.162	0.012	0.170
Passive rest	0.230	−0.082	0.617	−0.033	0.529	−0.043
Movement	0.857	0.012	0.069	−0.121	0.412	−0.056
Household activities	0.690	0.027	0.223	−0.081	0.005	−0.189
Occupational activity	0.866	0.012	0.660	−0.029	0.440	0.053
Activity by intensity						
Sedentary	0.060	−0.128	0.268	−0.074	0.234	−0.081
Light	0.841	0.014	0.153	−0.095	0.361	−0.062
Moderate	0.949	0.004	0.289	−0.071	0.044	−0.137
Vigorous	0.016	0.164	0.224	0.081	0.634	−0.033

Statistical significance for *p* < 0.05; Spearman’s rank—Spearman’s rank correlation coefficient; Prepandemic—tested before the pandemic (Dec 2019 to Mar 2020); COVID1—early in the pandemic (May to Sept 2020); COVID2—late in the pandemic (June to Sept 2021).

**Table 5:** Regression analysis—type of physical activity—women at risk of depression.

Variable	Compared Categories	Total Physical Activity [MET-h/Week]	Sports Activity and Physical Exercise [MET-h/week]	Passive Rest [MET-h/Week]	Movement [MET-h/Week]	Household Activities [MET-h/Week]	Occupational Activity [MET-h/Week]
		β (95% CI)	β (95% CI)	β (95% CI)	β (95% CI)	β (95% CI)	β (95% CI)
	COVID1 vs. Prepandemic						
Group	Model 1 ^#^	−20.971 (−65.801; 23.858)	1.095 (−3.300; 5.489)	−2.863 (−13.721; 7.994)	−11.166 (−21.642; −0.690) *	0.554 (−32.565; 33.647)	−8.591 (−22.332; 5.149)
	Model 2 ^##^	−17.881 (−60.896; 25.135)	1.608 (−2.934; 6.150)	−1.211 (−12.079; 9.656)	−8.459 (−19.070; 2.153)	−1.142 (−31.420; 29.135)	−8.676 (−22.885; 5.532)
	Model 3 ^###^	−23.103 (−65.360; 19.154)	1.446 (−3.084; 5.977)	−0.727 (−11.774; 10.320)	−8.486 (−19.192; 2.220)	−4.225 (−34.219; 25.770)	−11.111 (−25.191; 2.969)
	COVID2 vs. Prepandemic						
	Model 1 ^#^	−26.642 (−74.878; 21.595)	2.838 (−1.890; 7.567)	−10.163 (−21.845; 1.520)	−1.274 (−12.547; 9.998)	−13.304 (−48.940; 22.333)	−4.740 (−19.525; 10.045)
	Model 2 ^##^	−28.411 (−74.873; −18.050)	3.087 (−1.818; 7.993)	−9.094 (−20.832; 2.644)	−0.888 (−12.350; 10.574)	−16.373 (−49.076; 16.330)	−5.143 (−20.490; 10.203)
	Model 3 ^###^	−28.798 (−74.175; 16.580)	3.125 (−1.740; 7.990)	−8.709 (−20.571; 3.154)	−0.498 (−11.994; 10.999)	−16.492 (−48.701; 15.717)	−6.224 (−21.344; 8.895)

* *p* < 0.05; ^#^ Model 1 includes: group (COVID1/COVID2/Prepandemic (ref.)), EPDS ≥ 12 (score), IZZ (score); ^##^ Model 2 includes: group (COVID1/COVID2/Prepandemic (ref.)), EPDS ≥ 12 (score), IZZ (score), marital status (unmarried/married (ref.)), place of residence (village/city < 100,000/city > 100,000 (ref.)), number of deliveries (multiparous/primiparous (ref.)), education (primary or vocational or secondary/university (ref.)), planning the current pregnancy (yes/no (ref.)), complications in pregnancy (no/yes (ref.)); ^###^ Model 3 includes: group (COVID1/COVID2/Prepandemic (ref.)), EPDS ≥ 12 (score), IZZ (score), marital status (unmarried/married (ref.)), place of residence (village/city < 100,000/city > 100,000 (ref.)), number of deliveries (multiparous/primiparous (ref.)), education (primary or vocational or secondary/university (ref.)), planning the current pregnancy (yes/no (ref.)), complications in pregnancy (no/yes (ref.)), age (years), delivery at term (yes/no (ref.)), type of delivery (cesarean section/vaginal delivery (ref.)).

**Table 6:** Regression analysis—intensity of physical activity—women at risk of depression.

Variable	Compared Categories	Sedentary Physical Activity [MET-h/Week]	Light Physical Activity [MET-h/Week]	Moderate Physical Activity [MET-h/Week]	Vigorous Physiacal Activity [MET-h/Week]
		β (95% CI)	β (95% CI)	β (95% CI)	β (95% CI)
	COVID1 vs. Prepandemic				
Group	Model 1 ^#^	−4.973 (−12.939; 2.993)	−14.406 (−38.478; 9.665)	−1.030 (−26.411; 24.351)	−0.562 (−2.330; 1.206)
	Model 2 ^##^	−3.536 (−11.562; 4.489)	−14.792 (−38.535; 8.951)	0.846 (−23.171; 24.863)	−0.399 (−2.240; 1.442)
	Model 3 ^###^	−3.033 (−11.160; 5.094)	−17.558 (−41.288; 6.173)	−2.094 (−25.260; 21.071)	−0.418 (−2.260; 1.424)
	COVID2 vs. Prepandemic				
	Model 1 ^#^	−7.148 (−15.720; 1.423)	−23.656 (−49.557; 2.245)	3.944 (−23.366; 31.254)	0.219 (−1.684; 2.122)
	Model 2 ^##^	−6.324 (−14.992; 2.344)	−26.750 (−52.396; −1.105) *	4.146 (−21.795; 30.087)	0.518 (−1.471; 2.506)
	Model 3 ^###^	−6.006 (−14.733; 2.722)	−27.318 (−52.801; −1.836) *	4.004 (−20.872; 28.880)	0.522 (−1.456; 2.500)

*p < 0.05; ^#^ Model 1 includes: group (COVID1/COVID2/Prepandemic (ref.)), EPDS ≥ 12 (score), IZZ (score); ^##^ Model 2 includes: group (COVID1/COVID2/Prepandemic (ref.)), EPDS ≥ 12 (score), IZZ (score), marital status (unmarried/married (ref.)), place of residence (village/city < 100,000/city > 100,000 (ref.)), number of deliveries (multiparous/primiparous (ref.)), education (primary or vocational or secondary/university (ref.)), planning the current pregnancy (yes/no (ref.)), complications in pregnancy (no/yes (ref.)); ^###^ Model 3 includes: group (COVID1/COVID2/Prepandemic (ref.)), EPDS ≥ 12 (score), IZZ (score), marital status (unmarried/married (ref.)), place of residence (village/city < 100,000/city > 100,000 (ref.)), number of deliveries (multiparous/primiparous (ref.)), education (primary or vocational or secondary/university (ref.)), planning the current pregnancy (yes/no (ref.)), complications in pregnancy (no/yes (ref.)), age (years), delivery at term (yes/no (ref.)), type of delivery (cesarean section/vaginal delivery (ref.)).

## Data Availability

The data underlying this article will be shared on reasonable request to the corresponding author.

## Appendix A

In the analysis between the low-risk group for developing postpartum depression and activity, we observed a negative correlation between the EPDS score and total physical activity (Ppan: r = −0.235, *p* = 0.002), work (Ppan: r = −0.281, *p* < 0.001), and light work activity (Ppan: r = −0.282, *p* < 0.001) in prepandemic patients. In Cov1, we showed only an upward trend in depressive disorders and mobility-related activities (Cov1: r = 0.136, *p* = 0.060). In Cov2, it was noted that, despite not being at risk for the development of depressive disorders, passive activities contributed to increased EPDS scores. A similar relationship was observed in the case of sedentary activity (Ppan: r = 0.175, *p* = 0.023; Cov2: r = 0.189, *p* = 0.009) (Table A1).

**Table A1:** Spearman’s Rank Correlations between low risk of depressive disorders and physical activity—type and intensity of activity.

Lack of Risk of Postpartum Depressive Disorders						
Group	Prepandemic		COVID1		COVID2	
Parameters	*p*	Spearman’s Rank	*p*	Spearman’s Rank	*p*	Spearman’s Rank
Activity by type						
Total physical activity	0.002	−0.235	0.618	0.036	0.411	−0.60
Sports activity and physical exercise	0.268	0.086	0.657	−0.032	0.574	−0.041
Passive rest	0.217	0.096	0.659	−0.032	0.005	0.205
Movement	0.303	−0.080	0.060	0.136	0.145	−0.107
Household activities	0.060	−0.146	0.740	0.024	0.149	−0.106
Occupational activity	<0.001	−0.281	0.383	−0.063	0.862	0.013
Activity by intensity						
Sedentary	0.023	0.175	0.821	0.016	0.009	0.189
Light	<0.001	−0.282	0.602	0.038	0.405	−0.061
Moderate	0.123	−0.119	0.277	0.079	0.303	−0.076
Vigorous	0.522	−0.050	0.128	−0.110	0.083	0.127

Statistical significance for *p* < 0.05; Spearman’s rank—Spearman’s rank correlation coefficient; EPDS < 12; Prepandemic—tested before the pandemic (Dec 2019 to Mar 2020); COVID1—early in the pandemic (May to Sept 2020); COVID2—late in the pandemic (June to Sept 2021).

We also observed, independent of the study period, an increased risk of developing postpartum depressive disorders in patients correlated with decreased health behaviors. We observed a greater correlation in women studied during the Covid-19 pandemic (Cov1: r = −0.235, *p* < 0.001; Cov2: r = −0.237, *p* < 0.001) than before the pandemic (Ppan: r = −0.150, *p* = 0.024). Moreover, the increased risk of depressive disorders resulted in decreased positive mental attitudes, with the strongest correlation observed during the Cov2 (Ppan: r = 0.267, *p* < 0.001; Cov1: r = −0.295, *p* < 0.001; Cov2: r = −0.328, *p* < 0.001). Emotional problems during the pandemic also had a negative impact on eating habits (Cov1: r = −0.183, *p* = 0.005) and health practices (Cov1: r = −0.212, *p* = 0.001; Cov2: r = −0.209, *p* = 0.002, Table A2).

**Table A2:** Spearman’s Rank Correlations between depressive disorders and health behaviors.

Groups	Prepandemic		COVID1		COVID2	
Parameters	*p*	Spearman’s Rank	*p*	Spearman’s Rank	*p*	Spearman’s Rank
EPDS vs. IZZ	0.024	−0.150	<0.001	−0.235	<0.001	−0.237
EPDS vs. EH	0.316	−0.067	0.005	−0.183	0.080	−0.119
EPDS vs. PB	0.447	−0.051	0.233	−0.078	0.131	−0.103
EPDS vs. PMA	<0.001	−0.267	<0.001	−0.295	<0.001	−0.328
EPDS vs. HP	0.662	−0.029	0.001	−0.212	0.002	−0.209

Statistical significance for *p* < 0.05; Spearman’s rank—Spearman’s rank correlation coefficient; Prepandemic—tested before the pandemic (Dec 2019 to Mar 2020); COVID1—early in the pandemic (May to Sept 2020); COVID2—late in the pandemic (June to Sept 2021); EPDS—Edinburgh Postnatal Depression Scale; IZZ—Health Behavior Inventory (pol. Inwentarz Zachowań Zdrowotnych); EH—eating habits; PB—preventive behaviors; PMA—positive mental attitude; HP—health practices.

### Behavioral and Lifestyle Changes in Women during the COVID-19 Pandemic

Changes in the psychophysical state, e.g., level of stress, anxiety, and isolation, during the pandemic were examined using the author’s questionnaire. Increased anxiety and stress were observed in as many as 95.4% of Cov1 and 87.61% of Cov2. In Cov1, 91.57% were afraid of infection, while after the introduction of vaccines in 2021, this percentage decreased to 84.96% (Cov2). Up to 96.55% of pregnant women isolated at home in 2020, and 97.7% restricted social contact. Home isolation decreased to 81.86% a year later, with 90.27% still keeping with reduced social contact. In turn, 87.36% and 74.78% of women, (Cov1 and Cov2, respectively), believe that they decreased their physical activity due to the pandemic. Almost 74% of Cov1 accepted the ban on family-accompanying birth, which decreased to only 42.67% in Cov2 (Table A3).

**Table A3:** Changes in the psychophysical state and lifestyle of pregnant women due to the pandemic and related restrictions.

Parameters	COVID1 Group N = 261	COVID2 Group N = 226	* *p*
Increase in anxiety and stress compared to the period prior to the COVID-19 pandemic N/[%] Yes	249/95.40	198/87.61	0.002
Fear of coronavirus infection in pregnancy N/[%] Yes	239/91.57	192/84.96	0.022
Home isolation (e.g., another family member made purchases, no meetings with people other than household members) N/[%] Yes	252/96.55	185/81.86	<0.001
Limiting physical activity during pregnancy compared to the period before the COVID-19 pandemic N/[%] Yes	228/87.36	169/74.78	<0.001
Limiting social contact during pregnancy compared to the period before the COVID-19 pandemic N/[%] Yes	255/97.70	204/90.27	<0.001
Acceptance of the ban on family births due to the COVID-19 pandemic N/[%] Yes	73.95	42.67	<0.001

Statistical significance for *p* < 0.05; COVID1—early in the pandemic (May to Sept 2020); COVID2— late in the pandemic (June to Sept 2021); * based on chi-square test of independence.

Cov1 patients felt a greater degree of changes in their psychophysical state and lifestyle compared with women who gave birth a year later (Cov2, Table A4).

**Table A4:** The impact of the COVID-19 pandemic on changes in the lifestyle and psychophysical state of women giving birth during the pandemic.

Parameters	COVID1 Group	COVID2 Group	p *
	(N = 262) Me (Q1; Q3)	(N = 226) Me (Q1; Q3)	
To what degree did the current epidemiological situation increase your anxiety and stress compared to the period before the COVID-19 pandemic?	2 (2; 3)	2 (1; 3)	<0.001
How much did you fear coronavirus infection during pregnancy?	2 (2; 3)	2 (1; 3)	<0.001
To what degree did you isolate yourself at home after the outbreak of the COVID-19 pandemic (e.g., other family member did the shopping, no meetings with people other than household members)?	4 (3; 4)	2 (1; 3)	0.000
Due to the epidemiological situation, how much did you limit your physical activity during pregnancy compared to the period before the COVID-19 pandemic?	2 (1; 3)	1 (0; 2)	<0.001
Due to the epidemiological situation, how much did you limit your social activity during pregnancy compared to the period before the COVID-19 pandemic?	4 (3; 4)	2 (1; 3)	0.000

Statistical significance for *p* < 0.05; Me—median; Q1—lower quartile; Q3—higher quartile; COVID 1— early in the pandemic (May to Sept 2020); COVID2— late in the pandemic (June to Sept 2021); * based on U Mann Whitney test or chi-square test of independence.

Anxiety and stress due to the pandemic increased the risk for developing postpartum depression, especially during the later stage (r = 0.179, *p* = 0.004 vs. r = 0.265, *p* < 0.001). A risk factor for developing emotional problems in both groups was a high level of fear of Covid-19 infection (Cov1: r = 0.168, *p* = 0.007; Cov2: r = 0.257, *p* < 0.001) and, among women surveyed in 2021, the degree of home isolation (Cov2: r = 0. 165, *p* = 0.013, Table A5).

**Table A5:** Changes in lifestyle and psychophysical status and the risk of developing depressive disorders in women giving birth during the pandemic.

Postpartum Depressive Disorders				
Group	COVID1		COVID2	
Parameters	*p*	Spearman’s Rank	*p*	Spearman’s Rank
To what degree did the current epidemiological situation increase your anxiety and stress compared to the period before the COVID-19 pandemic?	0.004	0.179	<0.001	0.265
How much did you fear coronavirus infection during pregnancy?	0.007	0.168	<0.001	0.257
To what degree did you isolate yourself at home after the outbreak of the COVID-19 pandemic (e.g., other family member did the shopping, no meetings with people other than household members)?	0.749	−0.020	0.013	0.165
Due to the epidemiological situation, how much did you limit your physical activity during pregnancy compared to the period before the COVID-19 pandemic?	0.420	0.051	0.067	0.122
Due to the epidemiological situation, how much did you limit your social activity during pregnancy compared to the period before the COVID-19 pandemic?	0.334	−0.061	0.324	0.066

Statistical significance for *p* < 0.05; Spearman’s rank—Spearman’s rank correlation coefficient; COVID1— early in the pandemic (May to Sept 2020); COVID2— late in the pandemic (June to Sept 2021).

The level of fear of infection increased with the level of health behaviors (r = 0.149, *p* = 0.021) during the early pandemic, but no similar relationship was present a year later (r = 0.086, *p* = 0.204). Regardless of the pandemic stage, higher levels of health habits resulted in increased isolation at home (Cov1: r = 0.127, *p* = 0.05; Cov2: r = 0.224, *p* < 0.001). A similar correlation was noted in relation to social meetings (Cov1: r = 0.153, *p* = 0.018; Cov2: r = 0.207, *p* = 0.002). In both cases, a stronger relationship was observed in the Cov2 group (Table A6).

**Table A6:** Changes in lifestyle and psychophysical status and the level of general health habits in women giving birth during the pandemic.

General Health Habits				
Group	COVID1		COVID2	
Parameters	*p*	Spearman’s Rank	*p*	Spearman’s Rank
To what degree did the current epidemiological situation increase your anxiety and stress compared to the period before the COVID-19 pandemic?	0.310	0.066	0.682	−0.028
How much did you fear coronavirus infection during pregnancy?	0.021	0.149	0.204	0.086
To what degree did you isolate yourself at home after the outbreak of the COVID-19 pandemic (e.g., other family member did the shopping, no meetings with people other than household members)?	0.050	0.127	<0.001	0.224
Due to the epidemiological situation, how much did you limit your physical activity during pregnancy compared to the period before the COVID-19 pandemic?	0.216	0.080	0.864	0.012
Due to the epidemiological situation, how much did you limit your social activity during pregnancy compared to the period before the COVID-19 pandemic?	0.018	0.153	0.002	0.207

Statistical significance for *p* < 0.05; Spearman’s rank—Spearman’s rank correlation coefficient; COVID1—early in the pandemic (May to Sept 2020); COVID2—late in the pandemic (June to Sept 2021).

## References

[1] World Health Organization Coronavirus Disease (COVID-19) Pandemic. https://www.who.int/europe/emergencies/situations/covid-19.

[2] Duszyński J., Afelt A., Ochab-Marcinek A., Owczuk R., Pyrć K., Rosińska M., Rychard A., Smiatacz T. Zrozumieć COVID-19. https://informacje.pan.pl/informacje/materialy-dla-prasy/3111-zrozumiec-covid-19-opracowanie-zespolu-ds-covid-19-przy-prezesie-pan.

[3] Fundacja Rodzić po Ludzku Polskie Regulacje w Zakresie Opieki nad Kobietą w Ciąży, Porodzie i Po Porodzie w Czasie Pandemii SARS-CoV-2—Dotyczy Kobiet w Okresie Okołoporodowym. https://rodzicpoludzku.pl/aktualnosci/polskie-regulacje-w-zakresie-opieki-nad-kobieta-w-ciazy-porodzie-i-po-porodzie-w-czasie-pandemii-sars-cov-2-dotyczy-kobiet-w-okresie-okoloporodowym/.

[4] Serwis Ministerstwa Zdrowia i Narodowego Funduszu Zdrowia Odwiedziny u Dorosłych Pacjentów w Szpitalu. https://pacjent.gov.pl/aktualnosc/odwiedziny-u-doroslych-pacjentow-w-szpitalu.

[5] Ministerstwo Zdrowia Rozporządzenie Ministra Zdrowia z Dnia 12 Maja 2022 r. w Sprawie Odwołania na Obszarze Rzeczypospolitej Polskiej Stanu Epidemii. https://isap.sejm.gov.pl/isap.nsf/DocDetails.xsp?id=WDU20220001027.

[6] Ahmad M., Vismara L. (2021). The Psychological Impact of COVID-19 Pandemic on Women’s Mental Health during Pregnancy: A Rapid Evidence Review. Int. J. Environ. Res. Public Health.

[7] Akgor U., Fadıloglu E., Soyak B., Unal C., Cagan M., Temiz B.E., Erzenoglu B.E., Ak S., Gultekin M., Ozyuncu O. (2021). Anxiety, depression and concerns of pregnant women during the COVID-19 pandemic. Arch. Gynecol. Obstet..

[8] Moyer C.A., Compton S.D., Kaselitz E., Muzik M. (2020). Pregnancy-related anxiety during COVID-19: A nationwide survey of 2740 pregnant women. Arch. Womens Ment. Health.

[9] Puertas-Gonzalez J.A., Mariño-Narvaez C., Peralta-Ramirez M.I., Romero-Gonzalez B. (2021). The psychological impact of the COVID-19 pandemic on pregnant women. Psychiatry Res..

[10] Park S., Marcotte R.T., Staudenmayer J.W., Strath S.J., Freedson P.S., Chasan-Taber L. (2022). The impact of the COVID-19 pandemic on physical activity and sedentary behavior during pregnancy: A prospective study. BMC Pregnancy Childbirth.

[11] Ostacoli L., Cosma S., Bevilacqua F., Berchialla P., Bovetti M., Carosso A.R., Malandrone F., Carletto S., Benedetto C. (2020). Psychosocial factors associated with postpartum psychological distress during the COVID-19 pandemic: A cross-sectional study. BMC Pregnancy Childbirth.

[12] Liang P., Wang Y., Shi S., Liu Y., Xiong R. (2020). Prevalence and factors associated with postpartum depression during the COVID-19 pandemic among women in Guangzhou, China: A cross-sectional study. BMC Psychiatry.

[13] Nomura Y., Araki T. (2022). Factors influencing physical activity in postpartum women during the COVID-19 pandemic: A cross-sectional survey in Japan. BMC Womens Health.

[14] COVID-19 Mental Disorders Collaborators (2021). Global prevalence and burden of depressive and anxiety disorders in 204 countries and territories in 2020 due to the COVID-19 pandemic. Lancet.

[15] Bueno-Notivol J., Gracia-García P., Olaya B., Lasheras I., López-Antón R., Santabárbara J. (2021). Prevalence of depression during the COVID-19 outbreak: A meta-analysis of community-based studies. Int. J. Clin. Health Psychol..

[16] Taquet M., Holmes E.A., Harrison P.J. (2021). Depression and anxiety disorders during the COVID-19 pandemic: Knowns and unknowns. Lancet.

[17] Rondung E., Leiler A., Meurling J., Bjärtå A. (2021). Symptoms of Depression and Anxiety During the Early Phase of the COVID-19 Pandemic in Sweden. Front. Public Health.

[18] Maharlouei N., Keshavarz P., Salemi N., Lankarani K.B. (2021). Depression and anxiety among pregnant mothers in the initial stage of the Coronavirus Disease (COVID-19) pandemic in the southwest of Iran. Reprod. Health.

[19] Hessami K., Romanelli C., Chiurazzi M., Cozzolino M. (2020). COVID-19 pandemic and maternal mental health: A systematic review and meta-analysis. J. Matern. Fetal Neonatal Med..

[20] Lebel C., MacKinnon A., Bagshawe M., Tomfohr-Madsen L., Giesbrecht G. (2020). Elevated depression and anxiety symptoms among pregnant individuals during the COVID-19 pandemic. J. Affect. Disord..

[21] Wu Y., Zhang C., Liu H., Duan C., Li C., Fan J., Li H., Chen L., Xu H., Li X. (2020). Perinatal depressive and anxiety symptoms of pregnant women during the coronavirus disease 2019 outbreak in China. Am. J. Obstet. Gynecol..

[22] Kahyaoglu Sut H., Kucukkaya B. (2021). Anxiety, depression, and related factors in pregnant women during the COVID-19 pandemic in Turkey: A web-based cross-sectional study. Perspect. Psychiatr. Care.

[23] Papadopoulos A., Nichols E.S., Mohsenzadeh Y., Giroux I., Mottola M.F., Van Lieshout R.J., Duerden E.G. (2021). Depression in pregnant women with and without COVID-19. BJPsych Open.

[24] Yan H., Ding Y., Guo W. (2020). Mental Health of Pregnant and Postpartum Women During the Coronavirus Disease 2019 Pandemic: A Systematic Review and Meta-Analysis. Front. Psychol..

[25] Acheampong K., Pan X., Kaminga A.C., Wen S.W., Liu A. (2021). Risk of adverse maternal outcomes associated with prenatal exposure to moderate-severe depression compared with mild depression: A fellow-up study. J. Psychiatr. Res..

[26] Al Rawahi A., Al Kiyumi M.H., Al Kimyani R., Al-Lawati I., Murthi S., Davidson R., Al Maniri A., Al Azri M. (2020). The Effect of Antepartum Depression on the Outcomes of Pregnancy and Development of Postpartum Depression: A prospective cohort study of Omani women. Sultan Qaboos Univ. Med. J..

[27] Orsolini L., Valchera A., Vecchiotti R., Tomasetti C., Iasevoli F., Fornaro M., De Berardis D., Perna G., Pompili M., Bellantuono C. (2016). Suicide during Perinatal Period: Epidemiology, Risk Factors, and Clinical Correlates. Front. Psychiatry.

[28] Bonari L., Pinto N., Ahn E., Einarson A., Steiner M., Koren G. (2004). Perinatal risks of untreated depression during pregnancy. Can. J. Psychiatry.

[29] Deave T., Heron J., Evans J., Emond A. (2008). The impact of maternal depression in pregnancy on early child development. BJOG.

[30] Wang Y., Li H., Peng W., Chen Y., Qiu M., Wang J., Hao Q., Tu Y., Liu Y., Zhu T. (2020). Non-pharmacological interventions for postpartum depression: A protocol for systematic review and network meta-analysis. Medicine.

[31] García González J., Ventura Miranda M.I., Requena Mullor M., Parron Carreño T., Alarcón Rodriguez R. (2018). Effects of prenatal music stimulation on state/trait anxiety in full-term pregnancy and its influence on childbirth: A randomized controlled trial. J. Matern. Neonatal Med..

[32] Hall H.G., Cant R., Munk N., Carr B., Tremayne A., Weller C., Fogarty S., Lauche R. (2020). The effectiveness of massage for reducing pregnant women’s anxiety and depression; systematic review and meta-analysis. Midwifery.

[33] Uguz F., Ak M. (2021). Cognitive-behavioral therapy in pregnant women with generalized anxiety disorder: A retrospective cohort study on therapeutic efficacy, gestational age and birth weight. Braz. J. Psychiatry.

[34] Hu Y., Wang Y., Wen S., Guo X., Xu L., Chen B., Chen P., Xu X., Wang Y. (2019). Association between social and family support and antenatal depression: A hospital-based study in Chengdu, China. BMC Pregnancy Childbirth.

[35] Kołomańska D., Zarawski M., Mazur-Bialy A. (2019). Physical Activity and Depressive Disorders in Pregnant Women-A Systematic Review. Medicina.

[36] Kołomańska-Bogucka D., Mazur-Bialy A.I. (2019). Physical Activity and the Occurrence of Postnatal Depression-A Systematic Review. Medicina.

[37] Ekelöf K., Andersson O., Holmén A., Thomas K., Almquist Tangen G. (2021). Depressive symptoms postpartum is associated with physical activity level the year prior to giving birth—A retrospective observational study. Sex Reprod. Healthc..

[38] World Health Organization Physical Activity. https://www.who.int/news-room/fact-sheets/detail/physical-activity.

[39] The American College of Obstetricians and Gynecologists Exercise During Pregnancy. https://www.acog.org/womens-health/faqs/exercise-during-pregnancy.

[40] Petrov Fieril K., Glantz A., Fagevik Olsen M. (2015). The efficacy of moderate-to-vigorous resistance exercise during pregnancy: A randomized controlled trial. Acta Obstet. Gynecol. Scand..

[41] Fell D.B., Joseph K.S., Armson B.A., Dodds L. (2009). The impact of pregnancy on physical activity level. Matern. Child Health J..

[42] Cramp A.G., Bray S.R. (2009). A prospective examination of exercise and barrier self-efficacy to engage in leisure-time physical activity during pregnancy. Ann. Behav. Med..

[43] Atkinson L., De Vivo M., Hayes L., Hesketh K.R., Mills H., Newham J.J., Olander E.K., Smith D.M. (2020). Encouraging Physical Activity during and after Pregnancy in the COVID-19 Era, and beyond. Int. J. Environ. Res. Public Health.

[44] Hillyard M., Sinclair M., Murphy M., Casson K., Mulligan C. (2021). The impact of COVID-19 on the physical activity and sedentary behaviour levels of pregnant women with gestational diabetes. PLoS ONE.

[45] Gildner T.E., Laugier E.J., Thayer Z.M. (2020). Exercise routine change is associated with prenatal depression scores during the COVID-19 pandemic among pregnant women across the United States. PLoS ONE.

[46] Kołomańska-Bogucka D., Micek A., Mazur-Bialy A.I. (2022). The COVID-19 Pandemic and Levels of Physical Activity in the Last Trimester, Life Satisfaction and Perceived Stress in Late Pregnancy and in the Early Puerperium. Int. J. Environ. Res. Public Health.

[47] Krzepota J., Sadowska D. (2017). Pregnancy Physical Activity Questionnaire—Polish version (PPAQ-PL). Med. Og. Nauk. Zdr..

[48] Chasan-Taber L., Schmidt M.D., Roberts D.E., Hosmer D., Markenson G., Freedson P.S. (2004). Development and validation of a Pregnancy Physical Activity Questionnaire. Med. Sci. Sports Exerc..

[49] Krzepota K., Sadowska D., Sempolska K., Pelczar M. (2017). Measuring physical activity during pregnancy—Cultural adaptation of the Pregnancy Physical Activity Questionnaire (PPAQ) and assessment of its reliability in Polish conditions. Agric. Environ. Med..

[50] Silva-Jose C., Sánchez-Polán M., Barakat R., Gil-Ares J., Refoyo I. (2022). Level of Physical Activity in Pregnant Populations from Different Geographic Regions: A Systematic Review. J. Clin. Med..

[51] Steiner M., Yonkers K. (1999). Depresja u Kobiet.

[52] Cox J.L., Holden J.M., Sagovsky R. (1987). Detection of postnatal depression. Development of the 10-item Edinburgh Postnatal Depression Scale. Br. J. Psychiatry.

[53] Kossakowska K. (2013). Edinburgh postnatal depression scale—Psychometric properties and characteristic. Acta Univ. Lodz. Folia Psychol..

[54] Golec M., Rajewska-Rager A., Latos K., Kosmala A., Hirschfeld A., Molińska-Glura M. (2016). The assessment of postpartum mood disorders and its risk factors. Psychiatry.

[55] Maliszewska K., Świątkowska-Freund M., Bidzan M., Preis K. (2017). Screening for maternal postpartum depression and associations with personality traits and social support. A Polish follow-up study 4 weeks and 3 months after delivery. Psychiatr. Pol..

[56] Padmapriya N., Bernard J.Y., Liang S., Loy S.L., Shen Z., Kwek K., Godfrey K.M., Gluckman P.D., Chong Y.S., Saw S.M. (2016). Association of physical activity and sedentary behawior with depression and anxiety symptoms during pregnancy in a multiethnic cohort of Asian women. Arch. Womens Ment. Health.

[57] Claesson I.M., Klein S., Sydsjö G., Josefsson A. (2014). Physical activity and psychological well-being in obese pregnant and postpartum women attending a weight-gain restriction programme. Midwifery.

[58] Juczyński Z. (2012). Narzędzia Pomiaru w Promocji i Psychologii Zdrowia.

[59] Antosiak-Cyrak K.Z., Demuth A. (2019). A study of physical activity levels of pregnant women using the Polish version of Pregnancy Physical Activity Questionnaire (PPAQ-Pl). Ginekol. Pol..

[60] Krzepota J., Sadowska D., Biernat E. (2018). Relationships between Physical Activity and Quality of Life in Pregnant Women in the Second and Third Trimester. Int. J. Environ. Res. Public Health.

[61] Wojtyła C., Ciebiera M., Wojtyła-Buciora P., Janaszczyk A., Brzęcka P., Wojtyła A. (2020). Physical activity patterns in third trimester of pregnancy—Use of pregnancy physical activity questionnaire in Poland. Ann. Agric. Environ. Med..

[62] Muhaidat N., Fram K., Thekrallah F., Qatawneh A., Al-Btoush A. (2020). Pregnancy During COVID-19 Outbreak: The Impact of Lockdown in a Middle-Income Country on Antenatal Healthcare and Wellbeing. Int. J. Womens Health.

[63] Whitaker K.M., Hung P., Alberg A.J., Hair N.L., Liu J. (2021). Variations in health behaviors among pregnant women during the COVID-19 pandemic. Midwifery.

[64] Chrzan-Dętkoś M., Walczak-Kozłowska T. (2020). Antenatal and postnatal depression—Are Polish midwives really ready for them?. Midwifery.

[65] Guvenc G., Yesilcinar İ., Ozkececi F., Öksüz E., Ozkececi C.F., Konukbay D., Kok G., Karasahin K.E. (2021). Anxiety, depression, and knowledge level in postpartum women during the COVID-19 pandemic. Perspect. Psychiatr. Care.

[66] Spinola O., Liotti M., Speranza A.M., Tambelli R. (2020). Effects of COVID-19 Epidemic Lockdown on Postpartum Depressive Symptoms in a Sample of Italian Mothers. Front. Psychiatry.

[67] Ceulemans M., Foulon V., Ngo E., Panchaud A., Winterfeld U., Pomar L., Lambelet V., Cleary B., O’Shaughnessy F., Passier A. (2021). Mental health status of pregnant and breastfeeding women during the COVID-19 pandemic-A multinational cross-sectional study. Acta Obstet. Gynecol. Scand..

[68] Safi-Keykaleh M., Aliakbari F., Safarpour H., Safari M., Tahernejad A., Sheikhbardsiri H., Sahebi A. (2022). Prevalence of postpartum depression in women amid the COVID-19 pandemic: A systematic review and meta-analysis. Int. J. Gynaecol. Obstet..

[69] Chrzan-Dętkoś M., Walczak-Kozłowska T., Lipowska M. (2021). The need for additional mental health support for women in the postpartum period in the times of epidemic crisis. BMC Pregnancy Childbirth.

[70] Abujilban S., Mrayan L., Al-Obeisat S., Tanash M., Sinclair M., Kernohan W.G. (2022). Factors associated with antenatal depression in the Kingdom of Jordan during the COVID-19 pandemic. PLOS Glob. Public Health.

[71] Motrico E., Domínguez-Salas S., Rodríguez-Domínguez C., Gómez-Gómez I., Rodríguez-Muñoz M.F., Gómez-Baya D. (2022). The Impact of the COVID-19 Pandemic on Perinatal Depression and Anxiety: A Large Cross-sectional Study in Spain. Psicothema.

[72] Nakamura A., van der Waerden J., Melchior M., Bolze C., El-Khoury F., Pryor L. (2019). Physical activity during pregnancy and postpartum depression: Systematic review and meta-analysis. J. Affect. Disord..

[73] Baran J., Kalandyk-Osinko K., Baran R. (2022). Does Prenatal Physical Activity Affect the Occurrence of Postnatal Anxiety and Depression? Longitudinal Study. Int. J. Environ. Res. Public Health.

[74] Sánchez-Polán M., Franco E., Silva-José C., Gil-Ares J., Pérez-Tejero J., Barakat R., Refoyo I. (2021). Exercise During Pregnancy and Prenatal Depression: A Systematic Review and Meta-Analysis. Front. Physiol..

[75] van der Waerden J., Nakamura A., Pryor L., Charles M.A., El-Khoury F., Dargent-Molina P., EDEN Mother–Child Cohort Study Group (2019). Domain-specific physical activity and sedentary behavior during pregnancy and postpartum depression risk in the French EDEN and ELFE cohorts. Prev. Med..

[76] Davenport M.H., Meyer S., Meah V.L., Strynadka M.C., Khurana R. (2020). Moms Are Not OK: COVID-19 and Maternal Mental Health. Front. Glob. Womens Health.

[77] Silva-Jose C., Nagpal T.S., Coterón J., Barakat R., Mottola M.F. (2022). The ‘new normal’ includes online prenatal exercise: Exploring pregnant women’s experiences during the pandemic and the role of virtual group fitness on maternal mental health. BMC Pregnancy Childbirth.

[78] Zhang Y., Ma Z.F. (2021). Psychological responses and lifestyle changes among pregnant women with respect to the early stages of COVID-19 pandemic. Int. J. Soc. Psychiatry.

[79] Yıldırım F., Günaydın N., Alpaslan Arar M. (2022). Determination of Depression, Anxiety and Stress in Pregnancy During the COVID-19 Pandemic. Erciyes Med. J..

[80] Matvienko-Sikar K., Pope J., Cremin A., Carr H., Leitao S., Olander E.K., Meaney S. (2021). Differences in levels of stress, social support, health behaviours, and stress-reduction strategies for women pregnant before and during the COVID-19 pandemic, and based on phases of pandemic restrictions, in Ireland. Women Birth.

[81] Pope J., Olander E.K., Leitao S., Meaney S., Matvienko-Sikar K. (2021). Prenatal stress, health, and health behaviours during the COVID-19 pandemic: An international survey. Women Birth.

[82] Lobel M., Cannella D.L., Graham J.E., DeVincent C., Schneider J., Meyer B.A. (2008). Pregnancy-specific stress, prenatal health behaviors, and birth outcomes. Health Psychol..

[83] Oechsle A., Wensing M., Ullrich C., Bombana M. (2020). Health Knowledge of Lifestyle-Related Risks during Pregnancy: A Cross-Sectional Study of Pregnant Women in Germany. Int. J. Environ. Res. Public Health.

[84] Ghahremani L., Alipoor M., Amoee S., Keshavarzi S. (2017). Health Promoting Behaviors and Self-efficacy of Physical Activity During Pregnancy: An Interventional Study. Int. J. Women’s Health Reprod. Sci..

